# Chemical Analysis and Anthelmintic Activity Against *Teladorsagia Circumcincta* of Nordic Bark Extracts *In vitro*

**DOI:** 10.3389/fvets.2021.666924

**Published:** 2021-06-04

**Authors:** Spiridoula Athanasiadou, Marit Almvik, Jarkko Hellström, Eva Madland, Nebojsa Simic, Håvard Steinshamn

**Affiliations:** ^1^Animal and Veterinary Science, Scotland's Rural College, Edinburgh, United Kingdom; ^2^NIBIO Norwegian Institute of Bioeconomy Research, Ås, Norway; ^3^LUKE Natural Resources Institute Finland, Jokioinen, Finland; ^4^Department of Chemistry, Norwegian University of Science and Technology, Trondheim, Norway

**Keywords:** plant extract, condensed tannins, plant compound, anthelmintic, sheep, pine tree, proanthocynidins, bark

## Abstract

Helminth parasitic infections are common in small ruminants in Norway; infection is usually treated with anthelmintic drugs, but anthelmintic resistance is an increasing problem. It is necessary to identify strategies to reduce the use of anthelmintic drugs and mitigate the impact of anthelmintic resistance. Condensed tannin (CT)-rich forages have been shown to reduce the helminth burden in small ruminants, but these forages have limited cultivation potential in Scandinavia. A good source for CT in cold climatic regions may be the bark of several commercially utilized tree species. In the present study, we determined the content and characterized the type of CT in bark extracts of pine (*Pinus sylvestris* L.), spruce (*Picea abies* L.), and birch (*Betula pubescens*). Extracts of selected bark samples were tested for their anthelmintic efficacy against the ovine infectious nematode *Teladorsagia circumcincta*. Total CT content was higher in the bark from younger (10–40 years old) pine and spruce trees; it decreased with tree age in pine, whereas it remained relatively stable in the bark of spruce and birch. Pine trees consisted of 100% procyanidins, whereas prodelphinins were present in most spruce (4–17%) and all birch samples (5–34%). Our studies clearly showed that there is variation in the anthelmintic activity of water and acetone extracts of bark samples collected from various sites around Norway, as this was measured with two independent *in vitro* assays, the egg hatch and larvae motility assays. The anthelmintic activity of some extracts was consistent between the two assays; for example, extracts from the three samples with the highest CT content showed very high activity in both assays, whereas the extract from the sample with the lowest CT content showed the lowest activity in both assays. For other extracts, activity was not consistent across the assays, which could be attributed to the susceptibility of the different stages of the parasitic life cycle. We demonstrated that bark extracts from commercially used trees in Scandinavia have the potential to be used as alternatives to anthelmintics. Further work should focus on refining the associations between bark extracts and anthelmintic activity to identify the best strategies to reduce the input of anthelmintic drugs in livestock production systems.

## Introduction

Recent research has shown that there is a high treatment frequency with anthelmintic drugs in sheep lambs in Norway, particularly in coastal areas (1), which may be responsible for the development of anthelmintic resistance (2, 3). Strategies to prevent helminth infections are needed to reduce treatments with anthelmintics. Dietary inclusion of plants or feed supplements containing condensed tannins (CT) may be one such strategy, as CT have been shown to have anthelmintic activity (4). CT are complex flavonoid polymers widely distributed in higher plants and hardwood and softwood bark (5, 6). Many common CT-rich forages which have been investigated in other parts of the world, such as birdsfoot trefoil (*Lotus corniculatus*), sulla (*Hedysarum coronarium*), sericea lespedeza (*Lespedeza cuneata*), and sainfoin (*Onobrychis viciifolia*), have cultivation limitations in Norway (7). However, Nordic countries have a strong forest and sawmill industry, which produces a vast amount of bark as a by-product. Especially softwood bark (8), which is rich in CT, is disposed off as fuel or used in horticulture (9). Exploiting the potential use of bark as a feed supplement for ruminants would make the tree industry more economically viable, as well as provide a strategy to mitigate the impact of anthelmintic resistance for sustainable parasite control.

There are very few records of antiparasitic effects of bark and CT extracted from trees, but a study by Min and coworkers showed that dietary inclusion of bark from *Pinus taeda L*., a species found in the United States of America, improved animal performance and reduced fecal egg count and ruminal ammonia concentration in growing male goats (10). Additionally, Williams et al. (11) observed direct anthelmintic effects of CT from *Pinus sylvestris* bark against the parasitic nematode *Ascaris suum*, which is prevalent in pigs and humans. To our knowledge, there is no published evidence on the concentration and type of CT in bark from common tree species in Norway, although a recent study showed that there is variation in tannin production in cultures of cells originating from Nordic plants (12). In this study, we aimed to (1) quantify the concentration and determine the type of CT in fresh bark from logging sites and in samples from commercial sawmills and (2) test the anthelmintic activity of selected bark extracts *in vitro* against *Teladorsagia circumcincta*, a common abomasal parasitic nematode in sheep and goats.

## Materials and Methods

### Bark Sampling

Twenty-nine bark samples were collected for the study. Bark of Scots pine (*Pinus sylvestris* L., *n* = 8), Norway spruce (*Picea abies* L. *n* = 8), and Downey birch (*Betula pubescens* Ehrh., *n* = 6) were sampled from trees felled from January to April in 2013 at different locations in the municipality of Tingvoll. Samples were taken from the lower and upper parts of the tree trunk, and the age of the tree was estimated by tree-ring dating. Additional bark samples of Scots pine (three samples) and Norway spruce (four samples) were provided by the sawmills MøreTre AS (Surnadal) and Kjeldstad Sagbruk & Høvleri AS (Selbu), respectively. All bark samples were composed of both inner and outer bark (not separated). The samples were dried at 65°C for 48 h, cut into smaller pieces, and homogenized to pass a 0.5-mm screen using a rotor mill (Retsch Ultra Centrifugal Mill ZM 200) prior to extraction.

### Condensed Tannins Analysis

CT exist in both free and bound states in the bark (8). Free CT may be extracted with aqueous acetone, aqueous methanol, or alkaline hot water (13–17), whereas non-extractable (or bound) CT require chemical or enzymatic pre-treatment, e.g., thiolysis, to be released (5, 18).

#### Total CT Determination

**Figure 1:**
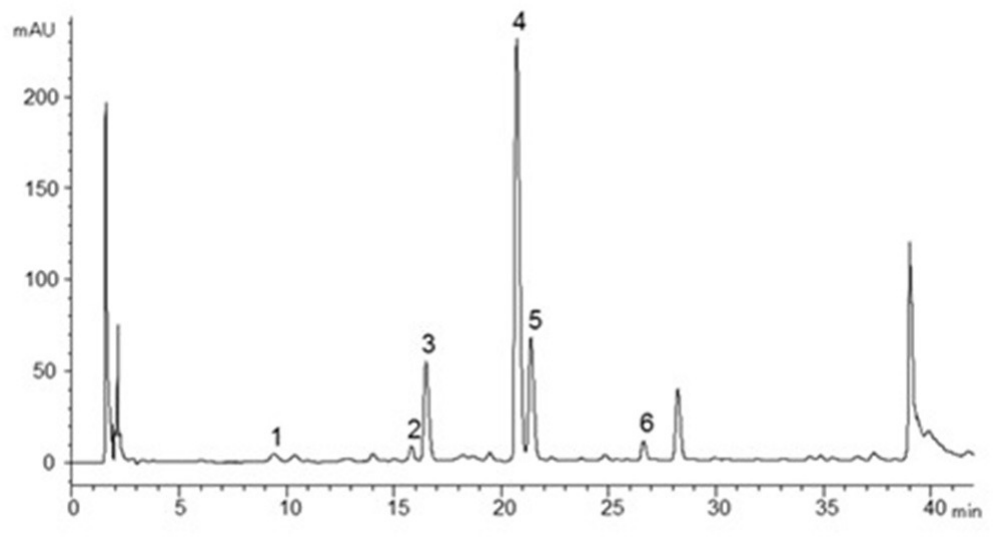
HPLC-PDA (λ = 280 nm) chromatogram of birch bark sample after thiolysis. Peaks are cysteaminyl thioethers of gallocatechin (1), epigallocatechin (2), catechin (3), and epicatechin (4), respectively. Peak 5 corresponds to catechin and peak 6 to epicatechin (pure/non-derivatized compounds).

All bark samples were analyzed for total CT content (free plus bound CT) by high-performance liquid chromatography (HPLC) coupled to a photodiode array (PDA) detector after thiolysis, that is, a depolymerization of the CT in the presence of a nucleophile (cysteamine) (19), at the Biotechnology and Food Research laboratory of LUKE Natural Resources Institute Finland. In short, 10 mg homogenized bark sample was weighed into an Eppendorf tube and 1 ml thiolysis reagent [3 g cysteamine + 56 ml methanol + 4 ml aqueous (37%) HCl] was added. The sample was put in a water bath at 65°C for 60 min and then cooled on ice for 5 min. The sample was homogenized using a Whirli mixer and filtrated (0.45 μm regenerated cellulose syringe filter) into an HPLC vial and analyzed immediately. During the thiolysis reaction, the CT B-type bonds were broken. The extension units were released as flavanol-cysteamine derivatives while the terminal units were released as underivatized free flavanol monomers. These units were determined individually with HPLC-PDA and summed into total CT content (18). The A-type bonds remained intact and produced dimers or higher oligomers, which could be distinguished from the monomeric flavanols by characteristic peaks during the chromatography. Each sample was analyzed in triplicate by HPLC-PDA on an Inertsil ODS-3 (GL Sciences Inc., Torrance, CA) reversed-phase column (150 × 4.0 mm i.d., 3 μm). The mobile phase consisted of (A) 50 mM phosphoric acid (aqueous), pH 2.5, adjusted by NaOH and (B) acetonitrile. Elution started isocratically with a constant flow of 5% B in A, 5 min, followed by 5–20% B in A, 5–35 min; 20–50% B in A, 35–45 min; and 50% B in A, 45–50 min. Separation was monitored by PDA at λ1 = 270 nm for (epi)gallocatechin) and λ2 = 280 nm for (epi)catechin and their cysteaminyl derivatives. Reference standards (gallocatechin, epigallocatechin, catechin, epicatechin, procyanidin B2, procyanidin A2) were purchased from Extrasynthese (Lyon, France) and used to set up external calibration curves for quantification. Cysteaminyl derivatives of (epi)gallocatechins were quantified using (epi)gallocatechin calibration curves. Cysteaminyl derivatives of (epi)catechins were quantified using the epicatechin cysteaminyl calibration curve generated from thiolyzed procyanidin B2. A-type dimers and their cysteaminyl derivatives were determined against procyanidin A2. A representative chromatogram is given in Figure 1. As the CT oligomers in the bark were cleaved into monomers during thiolysis, the nominal CT polymer sizes—or degree of polymerization (DP)—could not be determined, but the *average* degree of polymerization was calculated by dividing the total CT amount with the amount of terminal units.

#### Free CT Determination

Bark samples that were selected for *in vitro* anthelmintic testing were additionally analyzed for free CT content using a gravimetric ytterbium (Yb) precipitation method. Extractable CT were extracted with 70% acetone, according to a previously described method (20). Following the removal of acetone, the aqueous solution was washed three times with petroleum ether. Traces of solvent in the water phase were removed under vacuum before washing it three times with equal amounts of ethylacetate. Traces of ethylacetate in the aqueous phase were removed under vacuum. The remaining solution was separated into portions of 50 mL. Ytterbium(III) acetate (1 M, 2 ml) was added to the aqueous solution and stored overnight at 5°C. The next day, the stored solution was centrifuged at rpm = 3,590 for 10 min at 5°C. The formed pellet was washed and recentrifuged twice with 70 % acetone and once with pure acetone and dried under vacuum. The dry CT-ytterbium precipitation was weighed and heated to ashes at 800°C with a burner, above the oxidizing flame, until the black powder turned into gray ash. Before measuring the weight of the ash, the dish was cooled down to room temperature in a desiccator and the total amount of free CT was corrected for Yb(III) ash (21).

### Preparation of Bark Extracts for *in vitro* Anthelmintic Testing

Following the chemical analysis of the initial 29 bark samples collected for the study, 10 bark samples were selected based on CT content and type for *in vitro* anthelmintic testing. The aim was to cover all three tree species, so we included pine, spruce, and birch bark samples low and high in CT content and with variable proportions of PC and PD. Two types of bark extracts were prepared from these 10 bark samples to test the anthelmintic activity of CT, acetone, and water extracts. Seventy percent aqueous acetone solution is the solvent of choice for CT extraction; such CT extracts have been previously used in parasitological studies (21). To obtain those, 1 g of the dried bark sample was added to 20 ml of 70 % acetone and sonicated (probe sonicator) in ice for 20 sec. This was followed by stirring for 4 min. This procedure was followed five times, and the homogenate was filtered through Miracloth (Millipore, UK). The filtrate was then placed in a rotary evaporator until all acetone evaporated; the extract was resuspended in 20 ml of 1% dimethylsulfoxide (DMSO).

Hot water extraction was included in this study as the simplest extraction procedure and consequently one that may be favored by the industry. Although water is not the preferred solvent for CT extraction, hot water can extract free CT (22). To obtain the water extracts, 1 g of the dry bark sample was added to 20 ml of water, heated at 80°C, and stirred on a magnetic stirrer for 30 min. The homogenate was then filtered through Miracloth (Millipore, UK); the extract was resuspended in 20 ml of 1 % DMSO. Water and acetone extracts from each sample were tested with the assays described below. The two *in vitro* parasitological assays were selected, as they are routinely used to assess the anthelmintic efficacy of drugs and plant extracts.

### Egg Hatch Assays

An egg hatch assay (23) was used to test the anthelmintic efficacy of the selected bark extracts. *Teladorsagia circumcincta* eggs were isolated with a flotation technique (24) from freshly collected feces deriving from donor sheep monospecifically infected with the abomasal nematode. Following their isolation, eggs were washed with distilled water to remove any debris and quantified per ml of suspension. One hundred to 150 eggs were added in each well of 24-well plates, at volumes that did not exceed 500 μl; depending on the egg concentration in the suspension, distilled water was added to make up the appropriate volumes. An equal volume of the bark extract was added to each well. In the negative controls, distilled water was added instead of a bark extract. The plates were cultured at 20°C for 48 h. Hatching was stopped by adding helminthological iodine (10 g iodine, 50 g potassium iodine (KI), 100 ml deionized water in ¼ dilution) to the samples, and the numbers of eggs and first-stage larvae present were counted in each well. Each plant extract was tested in triplicate at 2% concentration (1 g of extract in 20 ml of 1% DMSO), and experiments were repeated. The hatching percentage was calculated as number of first stage larvae/number of first stage larvae + number of eggs per well.

### Larval Motility Assays

A high-throughput larval motility assay was used to test the anthelmintic efficacy of the selected bark extracts. The DP xCELLigence Real-Time Cell Analyzer, which measures electrical impedance-based signals across interdigitated microelectrodes integrated on the bottom of tissue culture e-plates, has previously been developed to diagnose anthelmintic resistance (13). Third-stage (infective larvae) *T. circumcincta* larvae were recovered from fecal cultures of monospecifically infected donor sheep after a 10-day incubation period at 20°C. Larval suspension [3,000 L3 per 100 μl of phosphate-buffered saline (PBS)] was added to the wells of E-plates, and the impedance was monitored every 15 sec, for 24 h at 37°C. All larvae used in the experiments were freshly produced (within 3 months of development to L3). At the end of this period, bark extracts were added in all wells at the 2 % concentration (1 g of extract in 20 ml of 1% DMSO) except the negative control wells, where 1% DMSO was added; these wells served as negative controls, and the expectation was that these larvae will be maintained alive until the end of the experiment. In addition to the negative controls, positive controls (dead larvae, which were frozen and maintained at −20°C for a month) were also included in three wells. The wells with dead larvae received bark extracts after 24 h, to enable the testing of our hypothesis (see below). Wells with no worms were also included as technical controls. All controls were present in all experiments. Impedance signals were monitored in total for 48 h (24 h prior to and 24 h post extract addition) before stopping the experiments. Each treatment was run in triplicate (technical replicate). Impedance data were analyzed and a motility index was calculated as described by Smout et al. (25). Motility index data were used for the statistical analysis as described below.

### Statistical Analysis

Statistical analyses on bark total CT concentration and degree of polymerization were carried out using a mixed-model procedure in SAS (Version 9.4; SAS Institute Inc., Cary, NC). The categorical variable tree Species (birch, pine and spruce) and the continuous variable tree age and their interaction were included as fixed effects, and the effect of Location was treated as random effect. LSmeans was used to compare “Species” means, and Tukey's multiple-comparison test was used to determine the significant (*P* < 0.05) difference between treatments. The “estimate” statement in the mixed-model procedure of SAS was used to obtain tree species-specific intercepts and slopes, and the Satterthwaite approximation was used to determine the denominator degrees of freedom for the test of effects.

All egg hatching experiments were duplicated, and the experiments were included in the model as a block. Egg hatching data were analyzed with one-way ANOVA, with treatment as factor. Larval motility data were averaged prior to (24 h) and following the addition of the bark extract (24 h). Motility data following the addition of the bark extract were analyzed with one-way ANOVA, with motility data prior to the addition of the bark extracts used as covariate. Bark extract type (water or acetone) was included in the model as a fixed factor. Each experiment had the following setup: alive larvae treated with water or acetone extract from the same sample, dead control larvae also treated with the two extracts from the same sample, alive controls, which were throughout left untreated, and no worm control wells (*n* = 3). Readings from the no-worm control treatment were not included in the statistical model as these were only included in the experiment as technical controls. Our null hypothesis was that the motility of the larvae exposed to bark extracts was significantly different (*P* < 0.05) than that of the dead control larvae. This was the most appropriate comparison as the environment was the same in these treatments (alive larvae treated with bark extracts and dead larvae treated with bark extracts).

## Results

### Condensed Tannins in the Bark Samples

The pine and spruce bark samples were composed of both inner and outer bark, and the homogenized bark from the older trees (>60 years old) had a higher outer/inner bark ratio and displayed a dark brown appearance, compared to the homogenized bark from the younger trees, which had a yellow appearance. Tree age did not affect color variation of the homogenized birch bark samples.

**Figure 2:**
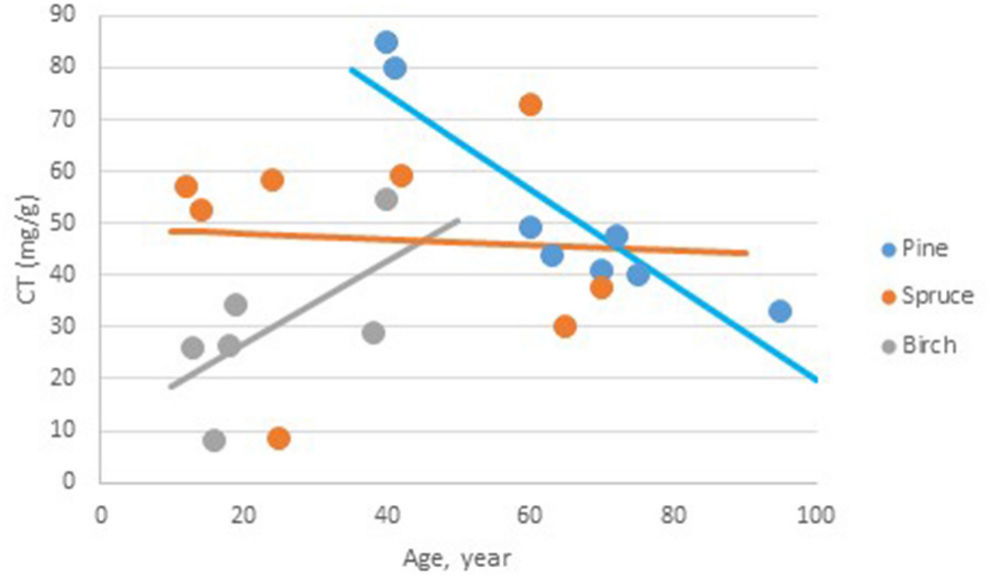
The relationship between age of the trees and the total content of condensed tannins (mg CT/g bark) in bark from Norwegian pine, spruce, and birch. Pine CT (g/kg) = 111.8 (SE 21.9, *p* < 0.001, df = 15.9)−0.92 (SE 0.328, *p* = 0.013, df = 16) * Age. Spruce CT (g/kg) = 49.1 (SE 11.49, *p* < 0.001, df = 14.1)−0.05 (SE 0.255, *p* = 0.842, df = 15.3) * Age. Birch CT (g/kg) = 10.7 (SE 15.64, *p* = 0.504, df = 16) + 0.80 (SE 0.591, *p* = 0.196, df=15.5) * Age.

The LSmeans of bark total CT content, that is free and bound CT determined with the chromatography method, was 72.2 (SE 8.7), 47.2 (SE 13.6), and 46.8 (SE 5.7) mg/g bark in pine, birch, and spruce, respectively. The Tukey test revealed that the content was significantly higher in pine than in spruce, while there were no significant differences between pine and birch and spruce and birch. There was a significant interaction between age of the trees and the species on the CT content (*P* = 0.047); the total CT content decreased [−0.92 (SE 0. 0.328) mg/g bark yearly] with age of the tree in pine (Figure 2). For the two other species, there were no significant effects of age on bark total CT content.

Only procyanidins (PC; catechin and epicatechin)—not prodelphinidins (PD; gallocatechin and epigallocatechin)—were detected in the pine bark samples. Procyanidins were also most predominant in spruce, but prodelphinidins were detected at an amount of 4–17% of total CT in 8 out of 12 spruce samples. Prodelphinidins were detected in all birch bark samples, constituting 5–34% of total CT content. Epicatechin was the predominant procyanidin in all species. The ratio of epicatechin:catechin was measured to 4:1 and 5:1 in pine and spruce, respectively, and 2:1 in birch. The average degree of CT polymerization was significantly higher in spruce (8.2 SE 0.38) than in birch (5.7 SE 0.44) and pine (6.6 SE 0.38) samples.

CT characteristics of a selection of the bark samples (corresponding to the 10 samples tested for anthelmintic effects) are displayed in Table 1. The bark CT monomers were predominantly linked by B-type interflavan bonds (i.e., single carbon–carbon bonds), but some A-type bonds (additional interflavonoid ether bond together with a common B-type bond, <1.3 %) were detected in pine samples (data not shown in Table 1). The average number of monomers in the CT oligomers, given as the average degree of polymerization (DP), was approximately 7–10 in spruce bark, five to seven in pine bark, and five to six in birch. The spruce bark from Selbu (sample ID 61) had one of the largest average polymer sizes and had a higher content of prodelphinidins (16.7% of CT) than the other spruce bark samples.

**Table 1:** Analysis of total CT in selected bark samples used for anthelmintic screening by high-performance liquid chromatography (HPLC) coupled to a photodiode array detector after thiolysis (*n* = 3).

**Species**	**ID no**.	**Location**	**Age (years)**	**Total CT**				
				**Conc. (mg/g)**	**SD (*n* = 3)**	**DP**	**Procyanidins (%)**	**Prodelphinidins (%)**
*Pinus sylvestris*	2	Tingvoll	40	85.1	1.1	5.3	100	0
	9	Tingvoll	70	40.8	0.7	6.5	100	0
	12	Tingvoll	41	79.8	1.4	7.2	100	0
	41^a^	Surnadal	NA	35.6	1.2	6.3	100	0
*Picea abies*	7	Tingvoll	42	59.4	2.6	7.5	94.4	5.6
	15	Tingvoll	60	72.7	0.6	7.2	100	0
	27	Tingvoll	25	8.4	0.4	10.8	100	0
	61^a^	Selbu	NA	26.1	0.3	10.1	83.3	16.7
*Betula pubescens*	3	Tingvoll	40	54.8	2.3	5.6	84.9	15.6
	52	Tingvoll	16	8.2	0.4	6.0	65.8	34.2

a *Samples from sawmills*.

*NA, not available*.

*Average degree of polymerization (DP) and content of procyanidins (% (epi)catechin) and prodelphinidins (% (epi)gallocatechin) are also reported*.

The proportion of CT that was extractable with aqueous acetone, that is, the free CT determined with gravimetric method, varied between 9 and 18 mg/g and constituted approx. 17–29% of the total CT in pine (*n* = 3). Free CT appeared to be higher in the younger trees compared to the older trees (15.6 mg free CT/g bark in 40-year-old trees vs. 11.7 mg free CT/g bark in a 70-year-old tree). The free CT concentration in spruce varied between 6 and 18 mg/g, constituting 11–35% of the total CT amount (*n* = 2). Free CT concentration was measured in a single birch sample from a 40-year-old tree and was estimated at 25 mg free CT/g bark.

### Egg Hatch Assays

The percentage of egg hatching under control conditions was consistently between 95 and 99%. Overall, incubation in acetone extracts resulted in average hatching of 11% and in water extracts of 20%, although this difference was not significant (*P* = 0.199). Incubation in either water or acetone bark extracts resulted in most cases in significantly reduced hatching compared to the controls (Table 2). Extracts from pine samples showed consistently high efficacy, with acetone extracts from most samples inhibiting hatching up to 100% (Table 2). Incubation in water and acetone extracts from birch 52 did not affect hatching whereas birch three extracts significantly reduced hatching compared to controls (*P* < 0.01), although the magnitude of the effect was not the same as in extracts from pine or spruce samples.

**Table 2:** Percentage of *T. circumcincta* egg hatching [*n* = 6 (3 reps × 2 experiments per extract)] following incubation in either water extracts or acetone extracts at 2 % concentration (1 g of extract in 20 ml of 1% DMSO) of bark samples for 48 h.

**Species**	**Id**	**Egg hatching (%)**	
		**Water extract**	**Acetone extract**
*Pinus sylvestris*	2	8	1
	9	1	0
	12	8	0
	41	2	1
SED		2.3	
*Picea abies*	7	11	1
	15	13	0
	27	0	1
	61	12	0
SED		1.4	
*Betula pubescens*	3	65	61
	52	96	99
SED		3.6	

*SED, standard error of difference*.

*Hatching of eggs incubated in water served as control, and hatching was consistently higher than 95%*.

### Larval Motility Assays

**Figure 3:**
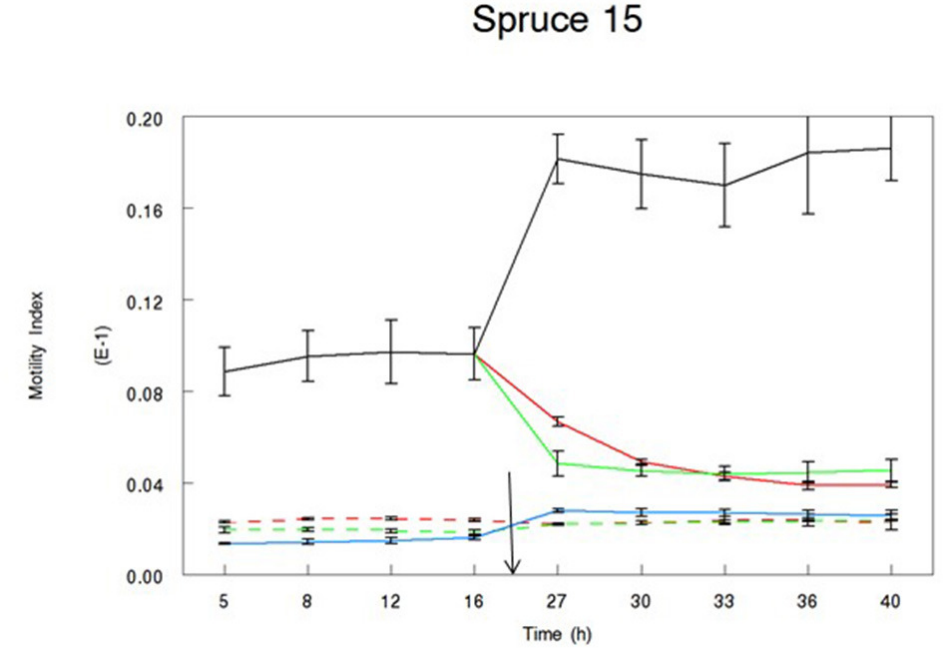
Motility Index of L3 *T. circumcincta* larvae incubated in bark extracts of spruce sample no. 15. Acetone (green lines) or water extract (red lines) reconstituted in 1% dimethyl sulfoxide (DMSO) was added in alive (solid line) or dead (dotted line) *T. circumcincta* L3 24 h in the experiment (black arrow). One percent DMSO was added in control wells, where control *T. circumcincta* L3 were maintained alive until the end of the trial (black line). No worm controls were also added in the experiment as technical controls (blue line). Bars indicate standard error (*n* = 3).

**Figure 4:**
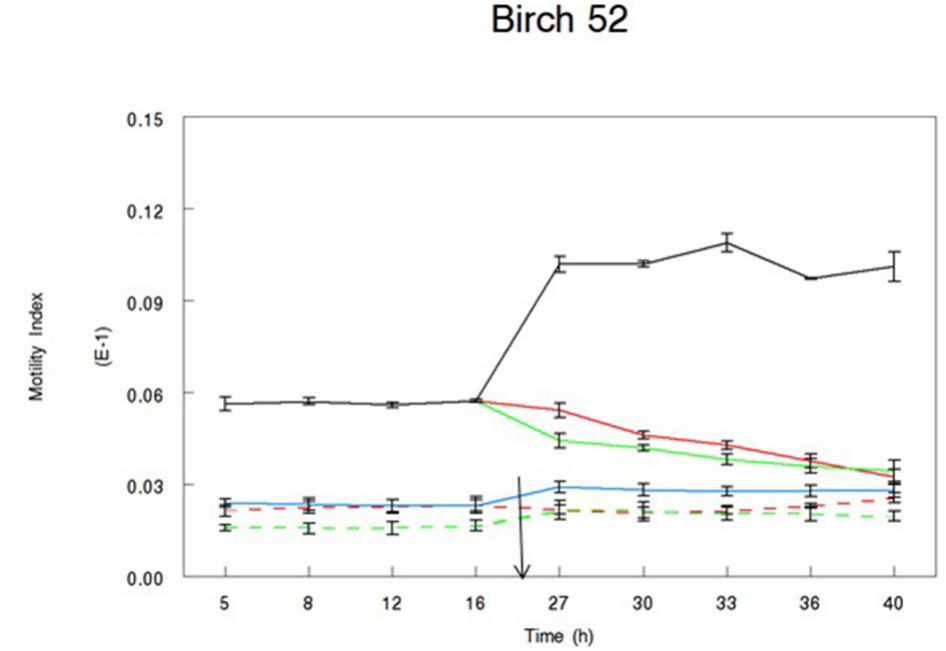
Motility Index of L3 *T. circumcincta* larvae incubated in bark extracts of birch sample no 52. Acetone (green lines) or water extract (red lines) reconstituted in 1% dimethyl sulfoxide (DMSO) was added in alive (solid line) or dead (dotted line) *T. circumcincta* L3 24 h in the experiment (black arrow). One percent DMSO was added in control wells, where control *T. circumcincta* L3 were maintained alive until the end of the trial (black line). No worm controls were also added in the experiment as technical controls (blue line). Bars indicate standard error (*n* = 3).

Throughout the duration of all experiments, the motility of alive control larvae was significantly higher than that of dead larvae (motility: 0.012 vs. 0.002, respectively; sed: 0.0002; *P* < 0.001). Our data show that the biological activity of the extracts was variable between samples and between extracts from the same samples (Table 3). In all experiments, motility of alive treated larvae (following the addition of the bark extract) was significantly different from that of alive control larvae. Table 3 shows the full set of data and all the treatments that allow the rejection of our hypothesis. Our hypothesis was rejected following the incubation in four acetone and five water extracts. The motility of larvae treated with acetone extracts from two pine samples (2 and 12) and two spruce samples (15 and 611) was reduced to the level of dead larvae. Similarly, the incubation of larvae in water samples from three pine samples (2, 12, and 41) and two spruce samples (7 and 611) resulted in their motility reduced to the level of dead larvae. None of the remaining of the extracts resulted in reduced motility of L3 to the level of dead larvae, although in some cases motility of L3 following the addition of the extract was much lower than that of alive controls (Figures 3, 4).

**Table 3:** Motility of *T. circumcincta* larvae (*n* = 3) following incubation in bark sample extracts at 2% concentration (1 g of extract in 20 ml of 1% DMSO).

**Species**	**ID no**.	**Acetone extract**		**Water extract**		**Alive controls**	**SED**	**Rejecting the null hypothesis^a^**	
		**Alive treated**	**Dead control**	**Alive treated**	**Dead control**	**Nontreated**		**Acetone extracts**	**Water extracts**
*Pinus sylvestris*	2	0.0047	0.0038	0.0038	0.0035	0.0089	0.0005	Yes	Yes
	9	0.0047	0.0024	0.0045	0.0019	0.0099	0.0009	No	No
	12	0.0040	0.0034	0.0041	0.0034	0.0095	0.0009	Yes	Yes
	41	0.0056	0.0025	0.0038	0.0030	0.0096	0.0007	No	Yes
*Picea abies*	7	0.0061	0.0005	0.0058	0.0006	0.0198	0.0019	No	No
	15	0.0047	0.0014	0.0055	0.0015	0.0187	0.0020	yes	Yes
	27	0.0044	0.0028	0.0033	0.0024	0.0067	0.0013	No	Yes
	61	0.0045	0.0015	0.0061	0.0010	0.0194	0.0015	Yes	No
*Betula pubescens*	3	0.0045	0.0025	0.0045	0.0026	0.0104	0.0004	No	No
	52	0.0043	0.0013	0.0047	0.0017	0.0107	0.0008	No	No

a *Null hypothesis was that the motility of the larvae exposed to bark extracts was significantly different to the dead control larvae*.

*SED, standard error of difference*.

*The means are adjusted for covariate (motility of worms prior to addition of bark extracts)*.

## Discussion

### Condensed Tannin Concentration in Bark Samples

In the present study, we determined the variation in the content of total and free CT and characterized the type of CT in the bark extracts of Nordic trees, namely, *Pinus sylvestris* L., *Picea abies* L., and *Betula pubescens*. We showed that tree age is associated with CT content in pine but not in spruce and birch. We quantified the anthelmintic efficacy of bark extracts against the ovine nematode *Teladorsagia circumcincta* and showed that some extracts have the ability to reduce egg hatching and larval motility to almost 100%, which confirms the potential of using bark extracts for parasite control in ruminants.

From the three tree species tested, pine samples showed a decline in the bark content of total CT with tree age. Considering that older trees have more outer bark than younger trees, the expectation is that the outer:inner bark ratio would increase with age. Previous evidence has shown that the outer bark contains lower CT levels than the inner bark, (8). Matthews et al. (8) observed a decline in free CT in pine bark with increasing tree age, due to CT in the dead outer bark being bound to lignocellulose. A small decrease in the content of free CT with age was also observed in our samples (from 16 to 11 mg free CT/g bark), although it is unclear whether the decrease with age in total CT was only attributable to the decrease in free CT.

Commonly, most CT in tree barks are oligomers of procyanidins, with epicatechin and catechin as the monomeric units, followed by prodelphinidins, constituted by gallocatechin and epigallocatechin units. Our pine bark samples contained only procyanidins, which is supported with current literature (6, 8, 18). However, nearly all spruce and birch bark samples also contained both procyanidins and prodelphinidins. Procyanidins have been previously reported in birch bark (8, 11, 18), whereas prodelphinidins have not, although *Betula* leaves are known to have a high level of prodelphinidins (9). Epicatechin was the most predominant procyanidin in our samples; the pine and spruce bark contained four to five times as much epicatechin as catechin, whereas birch bark contained two times more epicatechin than catechin. This is in agreement with data found in the literature (6, 8). Pine bark samples contained the highest CT levels (mean value of 5 % total CT) when compared to spruce and birch, and the levels are in agreement with the 5–8 % CT levels reported in Scots pine inner bark and 5 % in outer pine bark (8, 18).

### Anthelmintic Activity of Selected Extracts

Our studies clearly showed that there is variation in the anthelmintic activity of water and acetone extracts of bark samples collected from various sites around Norway, as this was measured with two independent assays. Our data support a previously expressed view that the anthelmintic activity of acetone extracts may not always be related to the CT content of these extracts alone, and this will require further investigation. Furthermore, we showed that different stages of nematode parasites may vary in their susceptibility to active compounds contained in extracts from bark samples.

Egg hatching was significantly affected by incubation in most acetone bark extracts. Although this activity may be attributed to CT, other compounds may also be partly responsible for the activity observed, particularly as not all samples tested here had high CT content. The results here agree with previous evidence where incubation in purified extracts from pine bark (*Pinus radiata*) originating from New Zealand resulted in significant reduction in egg hatching and migration ability of *T. circumcincta* (26). In this study, concentrations of total CT of about 2 mg/g resulted in a reduction of egg hatching by 80%. Apart from two extracts, the concentrations of CT contained in our acetone extracts exceeded those previously reported and may be the reason for the high magnitude of activity observed in our egg hatching experiments.

The two extracts with the lowest CT content tested here had a very different activity pattern. Incubation of eggs in spruce 27 extracts significantly reduced hatching compared to controls, whereas incubation in birch 52 extracts did not affect egg hatching. This activity difference could be attributed to the degree of CT polymerization in the two samples. CT present in spruce 27 sample had the highest degree of polymerization, which was not the case for birch 52 samples. Our data support previous evidence where polymer size appeared to have a positive correlation with anthelmintic activity (11, 27). The two samples also differ in their CT profiling, with spruce 27 having 100% PCs, whereas the content of PCs in birch 52 is lower. The evidence related to the efficacy of PCs vs. PDs has been scarce and rather conflicting. PC-rich extracts from pine trees were more effective against parasite development compared to PD-rich ones (28). On the other hand, previous studies showed that PDs may be more efficient in reducing larval exsheathment (29), feeding (30), and adult motility *in vitro* (30). It appears that the issue of monomer efficiency may be more complex than previously thought and may also be affected by other factors, such as total CT content and degree of polymerization. The possibility that other compounds may be responsible for the activity of spruce 27 sample cannot be disregarded.

The results from the larval motility assays showed that incubation of larvae in water and acetone extracts resulted in a reduction in motility, with variable intensity. In some cases, water extracts showed weaker activity compared to acetone extracts, such as extracts from the pine 611 sample. In other cases, acetone extracts showed weaker activity, such as extracts from pine 41 and spruce 27. The three samples with the most CT in the acetone extracts were pine 2, pine 12, and spruce 15. Interestingly, larvae incubated in both acetone and water extracts of these samples reduced their motility to the level of dead larvae. Although CT were not determined in the water extracts in the current study, previous evidence has shown that CT can be extracted with hot water (22). These samples had the highest concentration of CT (73–85 mg Ct/g bark), free CT (14–18 mg free CT/g bark), average degree of polymerization, and no PDs. Consequently, it is possible that the water extraction resulted in free CT extraction, which was responsible for the activity.

The anthelmintic activity of many extracts was consistent between the two assays. Extracts from the three samples with the highest CT content showed very consistent activity in both assays. Similarly, birch 59 samples with the lowest CT content did not show biological activity against any of the parasitic stages. For others, activity was not consistent across the assays. For instance, extracts from pine 9 and spruce 7 samples showed very strong activity against the eggs, but that was not the case for the larvae motility assay. This variation in the magnitude of activities in the two assays could be attributed to the stage of the parasitic life cycle (4). Not unexpectedly, L3 appeared to be the least affected stage as infective larvae are the most resistant form of the parasite during their life cycle.

These results clearly showed that certain extracts from tree bark samples originating from Norway have anthelmintic activity that varies in intensity *in vitro*. Some of the extracts had substantial biological activity *in vitro*, which if validated *in vivo* could be utilized to reduce the input of anthelmintic drugs in livestock production systems to reduce the development and mitigate the impact of anthelmintic resistance. Further work should focus on the identification of the best tree type and age at felling to achieve maximum biological activity from the bark. Refining the associations between bark extracts and anthelmintic activity *in vivo* is essential to identify the best strategies to reduce the input of anthelmintic drugs in livestock production systems.

## Data Availability Statement

The original contributions presented in the study are included in the article/supplementary material, further inquiries can be directed to the corresponding author/s.

## Author Contributions

HS initiated the study. HS, SA, NS, and MA designed the study. HS conducted the sampling and preparation of the bark. MA, NS, EM, and JH performed the condensed tannin analysis of the bark. SA performed the parasitological assays and analyzed the results. SA, MA, and HS drafted the manuscript. All authors read and approved the manuscript.

## Conflict of Interest

The authors declare that the research was conducted in the absence of any commercial or financial relationships that could be construed as a potential conflict of interest.

## References

[1] Domke A Chartier C Gjerde B. Worm control practice against gastro-intestinal parasites in Norwegian sheep and goat flocks. Acta Vet Scand. (2011) 53:29. 10.1186/1751-0147-53-29 21569497 PMC3118134

[2] Domke AVM Chartier C Gjerde B Höglund J Leine N Vatn S. Prevalence of anthelmintic resistance in gastrointestinal nematodes of sheep and goats in Norway. Parasitol Res. (2012) 111:185–93. 10.1007/s00436-012-2817-x 22290446 PMC3378835

[3] Domke AVM Chartier C Gjerde B Stuen S. Benzimidazole resistance of sheep nematodes in Norway confirmed through controlled efficacy test. Acta Vet Scand. (2012) 54:48. 10.1186/1751-0147-54-48 22932059 PMC3511810

[4] Hoste H Jackson F Athanasiadou S Thamsborg SM Hoskin SO. The effects of tannin-rich plants on parasitic nematodes in ruminants. Trends Parasitol. (2006) 22:253–61. 10.1016/j.pt.2006.04.004 16632404

[5] Torres JL Selga A. Procyanidin size and composition by thiolysis with cysteamine hydrochloride and chromatography. Chromatographia. (2003) 57:441–5. 10.1007/BF02492538

[6] Porter L. Condensed tannins. In: Rowe J, editor. Natural Products of Woody Plants. Berlin Heidelberg: Springer (1989). p. 651–90.

[7] Athanasiadou S Tzamaloukas O Kyriazakis I Jackson F Coop RL. Testing for direct anthelmintic effects of bioactive forages against Trichostrongylus colubriformis in grazing sheep. Vet Parasitol. (2005) 127:233–43. 10.1016/j.vetpar.2004.09.031 15710524

[8] Matthews S Mila I Scalbert A Donnelly DMX. Extractable and non-extractable proanthocyanidins in barks. Phytochemistry. (1997) 45:405–10. 10.1016/S0031-9422(96)00873-4

[9] Mahnert K-C. Availability and the condition of Norwegian bark resources. Bioforsk FOKUS. (2014) 9:18–22.

[10] Min BR Solaiman S Gurung N Behrends J Eun JS Taha E. Effects of pine bark supplementation on performance, rumen fermentation, and carcass characteristics of Kiko crossbred male goats. J Anim Sci. (2012) 90:3556–67. 10.2527/jas.2011-4931 22851241

[11] Williams AR Fryganas C Ramsay A Mueller-Harvey I Thamsborg SM Keiser J. Direct anthelmintic effects of condensed tannins from diverse plant sources against Ascaris suum. PLoS ONE. (2014) 9:e97053. 10.1371/journal.pone.0097053 24810761 PMC4014605

[12] Suvanto J Nohynek L Seppänen-Laakso T Rischer H Salminen JP Puupponen-Pimiä R. Variability in the production of tannins and other polyphenols in cell cultures of 12 Nordic plant species. Planta. (2017) 246:227–41. 10.1007/s00425-017-2686-8 28382519 PMC5522657

[13] Kähkönen MP Hopia AI Heinonen M. Berry phenolics and their antioxidant activity. J Agric Food Chem. (2001) 49:4076–82. 10.1021/jf010152t 11513713

[14] Rohr GE Meier B Sticher O. Analysis of procyanidins. Stud Nat Prod Chem. (2000) 21:497–570. 10.1016/S1572-5995(00)80013-7

[15] Bertaud F Tapin-Lingua S Pizzi A Navarrete P Petit-Conil M. Development of green adhesives for fibreboard manufacturing, using tannins and lignin from pulp mill residues. Cellul Chem Technol. (2012) 46:449–55.

[16] Chupin L Motillon C Charrier-El Bouhtoury F Pizzi A Charrier B. Characterisation of maritime pine (Pinus pinaster) bark tannins extracted under different conditions by spectroscopic methods, FTIR and HPLC. Ind Crops Prod. (2013) 49:897–903. 10.1016/j.indcrop.2013.06.045

[17] Kemppainen K Siika-aho M Pattathil S Giovando S Kruus K. Spruce bark as an industrial source of condensed tannins and non-cellulosic sugars. Ind Crops Prod. 52:158–68. 10.1016/j.indcrop.2013.10.009

[18] Hellström JK Mattila PH. HPLC determination of extractable and unextractable proanthocyanidins in plant materials. J Agric Food Chem. (2008) 56:7617–24. 10.1021/jf801336s 18672884

[19] Karonen M Liimatainen J Sinkkonen J. Birch inner bark procyanidins can be resolved with enhanced sensitivity by hydrophilic interaction HPLC-MS. J Sep Sci. (2011) 34:3158–65. 10.1002/jssc.201100569 21998029

[20] Hagerman AE Hagerman AE. Tannin Chemistry Handbook. Livro. (2002) 116.

[21] Stringano E Gea A Salminen JP Mueller-Harvey I. Simple solution for a complex problem: proanthocyanidins, galloyl glucoses and ellagitannins fit on a single calibration curve in high performance-gel permeation chromatography. J Chromatogr A. (2011) 1218:7804–12. 10.1016/j.chroma.2011.08.082 21930278

[22] Bianchi S Kroslakova I Janzon R Mayer I Saake B Pichelin F. Characterization of condensed tannins and carbohydrates in hot water bark extracts of European softwood species. Phytochemistry. (2015) 120:53–61. 10.1016/j.phytochem.2015.10.006 26547588

[23] Von Samson-Himmelstjerna G Coles GC Jackson F Bauer C Borgsteede F Cirak VY. Standardization of the egg hatch test for the detection of benzimidazole resistance in parasitic nematodes. Parasitol Res. (2009) 105:825–34. 10.1007/s00436-009-1466-1 19452165

[24] Christie M Jackson F. Specific identification of strongyle eggs in small samples of sheep faeces. Res Vet Sci. (1982) 32:113–7. 10.1016/S0034-5288(18)32448-2 7201151

[25] Smout MJ Kotze AC Mccarthy JS Loukas A. A novel high throughput assay for anthelmintic drug screening and resistance diagnosis by real-time monitoring of parasite motility. PLoS Negl Trop Dis. (2010) 4:e885. 10.1371/journal.pntd.0000885 21103363 PMC2982823

[26] Molan A-L. Effect of purified condensed tannins from pine bark on larval motility, egg hatching and larval development of Teladorsagia circumcincta and Trichostrongylus colubriformis (Nematoda: Trichostrongylidae). Folia Parasitol (Praha). (2014) 61:371–6. 10.14411/fp.2014.036 25185408

[27] Novobilský A Stringano E Hayot Carbonero C Smith LMJ Enemark HL Mueller-Harvey I. *In vitro* effects of extracts and purified tannins of sainfoin (Onobrychis viciifolia) against two cattle nematodes. Vet Parasitol. (2013) 196:532–7. 10.1016/j.vetpar.2013.03.024 23639199

[28] Dhakal S Meyling NV Williams AR Mueller-Harvey I Fryganas C Kapel CMO. Efficacy of condensed tannins against larval Hymenolepis diminuta (Cestoda)*In vitro* and in the intermediate host Tenebrio molitor (Coleoptera) *In vivo*. Vet Parasitol. (2015) 207:49–55. 10.1016/j.vetpar.2014.11.006 25468673

[29] Brunet S Hoste H. Monomers of condensed tannins affect the larval exsheathment of parasitic nematodes of ruminants. J Agric Food Chem. (2006) 54:7481–7. 10.1021/jf0610007 17002411

[30] Desrues O Fryganas C Ropiak HM Mueller-Harvey I Enemark HL Thamsborg SM. Impact of chemical structure of flavanol monomers and condensed tannins on *in vitro* anthelmintic activity against bovine nematodes. Parasitology. (2016) 143:444–54. 10.1017/S0031182015001912 26888630 PMC4800716

